# Adverse Childhood Experiences (ACEs) in Specific Vulnerable Developmental Periods Can Increase the Likelihood of Chronic Pain in Adulthood—Results from a Cross-Sectional Study

**DOI:** 10.3390/diagnostics15070839

**Published:** 2025-03-25

**Authors:** David Riedl, Christina Kirchhoff, Ulrich T. Egle, Tobias Nolte, Michael Tschuggnall, Gerhard Rumpold, Wilhelm Kantner-Rumplmair, Vincent Grote, Michael J. Fischer, Astrid Lampe

**Affiliations:** 1 Ludwig Boltzmann Institute for Rehabilitation Research, 1140 Vienna, Austria; 2 Department of Psychiatry, Psychotherapy, Psychosomatics and Medical Psychology, University Hospital of Psychiatry II, Medical University of Innsbruck, 6020 Innsbruck, Austria; 3 Psychiatric Clinic Sanatorium Kilchberg, 8802 Kilchberg/ZH, Switzerland; 4 Anna Freud National Centre for Children and Families, London N1 9JH, UK; 5 Research Department of Clinical, Educational and Health Psychology, University College London, London WC1N 3AR, UK; 6 Evaluation Software Development (ESD), 6020 Innsbruck, Austria; 7 Department of Orthopaedics and Trauma, Medical University of Graz, 8036 Graz, Austria; 8 Rehabilitation Center Montafon, 6780 Schruns, Austria; 9 Rehabilitation Center Kitzbühel, 6370 Kitzbühel, Austria

**Keywords:** adverse childhood experiences, ACEs, chronic pain, childhood trauma, traumatization, machine learning

## Abstract

**Background:** Adverse childhood experiences (ACEs) have been identified as a major risk factor for physical and mental ill health in adulthood. So far, no studies have investigated whether ACEs during sensitive periods in infant development are associated with a higher likelihood of chronic pain (CP) in adulthood. **Methods**: Patients of the University Hospital of Innsbruck (Austria) completed questionnaires retrospectively assessing their ACEs as well as their current levels of CP, psychological distress, and trauma-related symptoms in this cross-sectional study. Dose-dependent associations of ACEs with CP were investigated through binary logistic regressions. To detect vulnerable developmental periods, a machine learning grid search was applied. **Results**: A total of *n* = 2577 patients were included in the analyses, with 31.5% reporting CP. Polytraumatization (i.e., four or more types of ACEs) was associated with a threefold increase for CP (OR: 3.01; 95% CI: 2.16–4.19; *p* < 0.001). The machine learning approach revealed maltreatment between 10 and 12 years to be the most predictive of CP (Ada Boost Classifier). **Discussion:** CP is a highly prevalent symptom among hospital patients and is clearly associated with ACEs. This is the first study to present evidence for a timing-dependent association of ACEs with CP. Early identification and appropriate psychosocial support for patients with ACEs is a crucial task for healthcare professionals.

## 1. Introduction

Chronic pain (CP) in adult life is a widespread and potentially disabling experience: approximately 12–14% of the general public in Europe and 19% of the U.S. general population report CP [1,2,3,4], and 7–14% suffer from moderately severe disabling CP [1,3]. CP is one of the leading causes worldwide for “years lived with disability” [5] and also has a severe socio-economic impact: in Europe, the mean annual costs associated with CP range between 3 and 10% of the gross domestic product [6].

Prior research has established strong links between depression, anxiety, catastrophizing, and traumatic experiences in individuals with chronic pain [7,8,9]. Depression and anxiety are frequent comorbidities in patients with CP and have been found to often exacerbate their pain perception and simultaneously reduce their quality of life [10]. Catastrophizing—which is defined as a maladaptive cognitive–emotional response characterized by an exaggerated negative orientation toward pain stimuli—may increase both pain intensity and psychological distress in the form of a mediating variable [11]. Additionally, traumatic experiences, particularly adverse childhood experiences (ACEs), can act as distal risk factors that heighten vulnerability to chronic pain in adulthood [12].

In the last decades, a growing body of research has shown a relationship between so-called “adverse childhood experiences” (ACEs) and CP in adulthood. ACEs refer to various types of physical and/or emotional abuse, neglect, or exploitation in children and adolescents [13]. While the original ACE study was a milestone in childhood trauma research, its initial framework did not encompass certain forms of abuse that were later found to be highly influential. Less investigated forms of ACEs include peer abuse or witnessing interparental violence and violence against siblings [14,15]. Several studies have reported ACEs (mainly sexual and physical abuse and emotional neglect) to be a risk factor leading to severe CP in adulthood [9,16,17]. While some research has linked specific *types of abuse* to different forms of chronic pain (e.g., sexual abuse with chronic pelvic pain) [18], other research has suggested a potential *dose-dependent effect* of a cumulative allostatic load via ACEs for some forms of CP experiences in adulthood: the number of different ACEs appears to be a more relevant risk factor for CP than the specific type of trauma [19,20].

Although the early onset and long duration of maltreatment have been regarded as greater risks for morphological neuronal change [21], some researchers have suggested that this might be an oversimplification. Childhood trauma can influence neurodevelopment through different interconnected pathways, including dysregulation of the hypothalamic–pituitary–adrenal (HPA) axis, heightened neuroinflammatory responses, and disruptions in neural connectivity [22,23]. These mechanisms may contribute to the onset of CP by altering brain maturation, impairing autonomic regulation, and disrupting the balance between excitatory and inhibitory neural processes [24]. Other important components which may play a critical role in the development of CP include immune system activation and metabolic alterations [25,26]. The impact of childhood trauma on CP therefore likely involves a complex interplay between neural, endocrine, and inflammatory pathways.

More recently, an additional focus was put on specific sensitive periods in infant brain development [27,28,29] which may be associated with mental disorders in adulthood [30,31,32]. Specifically, childhood maltreatment was identified as a potent risk factor with marked effects on morphology, function, and circuitry which may lead to atypical brain development [33]. The maturation of the pediatric brain is marked by enhanced connectivity between distinct brain regions, the specialization of neural circuits influenced by environmental factors, and an evolving equilibrium between the earlier-developing limbic system and the later-maturing prefrontal cortex, which continues to undergo significant transformation into the third decade of life [33,34,35]. The development of brain regions and fiber tracts follows age-related trajectories, with specific time frames during which they may be particularly vulnerable to environmental influences [28,30,36,37]. This would suggest that the developmental period (“*timing*”) of maltreatment is a key predictive factor of later impairment. Although a substantial body of research supports this hypothesis, a recent review indicated that no scientific consensus has yet been reached regarding a specific single period or multiple periods of heightened vulnerability [38]. In addition, so far, no study has investigated whether specific sensitive periods, which may be linked to a greater risk for the development of CP, can be identified [39].

The aim of the present study was to investigate a potential timing-dependent relationship between ACEs and CP in a cross-sectional sample of general hospital patients. This study employs a broad definition of CP, defining it as persistent or frequently recurring pain lasting at least six months, assessed through a self-reported dichotomous item. Additionally, an expanded conceptualization of ACEs—which also included peer abuse and witnessing abuse—was used to provide a more comprehensive understanding of their association with CP. Specifically, we hypothesized that specific vulnerable periods of development associated with a higher likelihood of CP can be identified.

## 2. Materials and Methods

### 2.1. Sample and Procedure

In this cross-sectional, observational study, inpatients and outpatients from seven departments at the University Hospital of Innsbruck (Otolaryngology, Trauma Surgery, Neurosurgery, Neurology, Gynecology, Internal Medicine, Radiology) were included between October 2015 and March 2017. Departments used for recruiting had to meet two criteria: the heads of departments agreed, and the on-site waiting periods in the respective departments were sufficiently long for patients to have time to complete the questionnaire. To minimize selection bias by the well-documented association between psychiatric disorders and ACEs, we did not include patients from psychiatric wards. The rationale was to investigate the relationship between ACEs and CP in a sample not preselected for psychiatric comorbidities, thereby providing a broader perspective on this association in a general hospital population. For a period of three months (per department), trained undergraduate psychology and medical students approached patients in waiting areas four mornings per week at the hospital. Patients were informed about the study, and those who gave oral and written consent completed the set of paper-and-pencil questionnaires on their own; questionnaires were then collected by the students. All patients received a handout with information about the study and were offered psychological support should they experience distress as a result of the questionnaires. Moreover, staff at the participating departments were informed about the study prior to its start and were instructed to pay attention to potentially distressed patients. The study design was in accordance with the Declaration of Helsinki (1964) and its later amendments and was approved by the research ethics committee of the Medical University of Innsbruck (AN2015-0175 351/4.18).

### 2.2. Measures

#### 2.2.1. Maltreatment and Abuse Chronology of Exposure (MACE) Scale

Adverse childhood experiences (ACEs) were assessed with the German version of the Maltreatment and Abuse Chronology of Exposure Scale (KERF; [40,41]). The questionnaire conceptually expands the original ACE framework by incorporating additional, previously underexplored forms of ACEs. It consists of 75 items that retrospectively assess the severity of exposure to ten types of maltreatment during each year of childhood and adolescence up to age 18. For each year separately, participants are asked to endorse whether maltreatment occurred during that particular year of their life or not. Thus, onset and cumulative exposure are measured. The items were grouped into seven ACE clusters: emotional abuse (verbal or non-verbal), physical abuse, neglect (emotional or physical), witnessing violence (between parents or towards siblings), physical peer abuse, emotional peer abuse, and sexual abuse. Clinical cut-off values for all subscales are available, and thus patient reports can be coded as metric (i.e., number of reported abuses) or dichotomized (i.e., above cut-off) data. The MACE scale provides an overall severity score and multiplicity score (number of types of maltreatment experienced) and has good test–retest reliability and validity [40,41]. The internal consistency for the MACE total score was excellent (*α* = 0.92).

#### 2.2.2. Essener Trauma Inventory (ETI)

To assess trauma-related symptoms, the Essener Trauma Inventory (ETI) was used. The ETI was designed to assess various aspects of traumatic events and allows for the classification of post-traumatic disorders according to DSM-IV criteria. The ETI consists of a trauma checklist (18 dichotomous items) and an assessment of trauma-related symptoms (23 items rated on a four-point Likert scale), followed by 5 items assessing functional impairment. Based on the trauma-related symptoms, a total score (range: 0 to 69) can be calculated, with higher values indicating more trauma-related distress. Values > 16 on the ETI total score indicate a notable distress level and values > 27 indicate a clinically relevant level of PTSD symptoms. Good internal consistency and validity have been reported for the total score and four subscales [42,43]. An excellent internal consistency (*α* = 0.94) for the ETI total score was found in our sample.

#### 2.2.3. Brief Symptom Inventory (BSI-18)

Psychological distress was assessed with the Brief Symptom Inventory (BSI-18) [44], which consists of 18 items rated on a four-point Likert scale (from “not at all” to “very often”). Gender-specific cut-off scores for the German BSI-18 global score are given and a good internal consistency for the total score (*α* = 0.89) has been reported [45].

#### 2.2.4. Pain Assessment

Presence of CP was assessed with a single dichotomized item (yes/no). CP was defined as permanent or frequently recurring pain for at least six month. Additionally, using a four-point scale from “not at all” (0) to “strongly” (4), patients rated how strongly they felt impaired by their pain.

### 2.3. Statistical Procedure

Sample characteristics and the prevalence of ACEs in the sample are shown by descriptive statistics. Analyses were limited to individuals with complete data for the MACE and pain assessment. Associations among ACEs and pain, psychological distress, or trauma-related symptoms were tested with independent-sample *t*-tests and Pearson correlation coefficients. To investigate whether patients with and without CP reported different maltreatment patterns, repeated-measure analyses of variance (ANOVAs) were calculated with the eighteen ACE sum scores as independent variables and CP (dichotomized: yes vs. no) as the dependent variable. Effect sizes of *η*^2^ = 0.01 and *d* = 0.3 were considered small, while *η*^2^ = 0.06 and *d* = 0.5 indicated a medium effect, and *η*^2^ = 0.14 and *d* = 0.8 indicated a large effect. For 2 × 2 contingency tables, ϕ–values (0.1 = small effect, 0.3 = medium effect, and 0.5 = large effect) are reported, while Cramer’s V is given for contingency tables exceeding 2 × 2 [46,47].

To assess the *dose-dependent effect* of ACEs (i.e., severity), a binary logistic regression analysis was performed. In line with previous research [14], the ACE frequency was grouped into three sub-samples (0 ACEs, 1–3 ACEs, ≥4 ACEs) and was entered as a categorial variable with 0 ACEs as the reference group.

The *type-dependent* effect was also evaluated using a binary logistic regression analysis, in which all six assessed forms of ACEs were added as independent variables and CP in adulthood was added as the dependent variable. Odds ratios (ORs) with 95% confidence intervals (CI) are shown.

To detect specific *vulnerable developmental periods*, machine learning was applied. Thereby, we tested several different feature groups and algorithms using an extensive grid search. In the first step, the eighteen items indicating abuse at each year were added separately as predictors for chronic pain in the form of metric variables (i.e., number of reported maltreatment experiences per year) as well as dichotomized variables (i.e., any maltreatment reported at the age vs. no maltreatment reported at the age). Subsequently, we also grouped the age variables with group sizes ranging from two to six. For example, with a group size of three, we computed a combined value for three consecutive years each, e.g., 0–2, 3–5, 6–8, etc. With respect to algorithms, we relied on commonly used techniques, including Support Vector Machines (SVMs), Random Forest, Extra Trees, Ada Boost Classification, Gradient Boosting, and Linear Discriminant Analysis (LDA). To compare the performances of the computed models, we computed a baseline model, which always predicts the majority as seen in the data set (i.e., no pain). As we intended for the machine learning models to not only predict a final dichotomized outcome (pain/no pain) but also the individual influences of the respective ages/age groups, we additionally extracted the respective feature importances of the computed models. To understand the given data, we trained the machine learning model using the F1 score, which is a common metric used to quantify the accuracy of a model. It is computed as the harmonic mean between the precision (e.g., if the model predicts “abuse at age 7”, how many times is it correct?) and recall (e.g., out of all existing abuses at age 7 in the data set, how much of them did the model find?). The final F1 score is then computed by averaging the individual results for all age groups, where we rely on the “weighted” variant incorporating the respective group sizes when calculating the average. The relative predictive value of each age class is displayed as a value of importance, ranging from 0 to 100%.

For descriptive purposes, age was clustered into three groups: 1–6 years (pre-school age), 7–12 years (latency school age), and 13–18 years (adolescence). These age clusters are in accordance with prior studies [48,49] and established definitions of developmental stages. Since the MACE scale gives patients the possibility to indicate multiple timepoints of abuse, patients could potentially be placed in all three age groups. *p*-values < 0.05 (two-sided) were considered statistically significant. Statistical analyses were performed with IBM SPSS Statistics, version 21 (IBM Corporation, Armonk, NY, USA).

## 3. Results

Of all the *n* = 3220 approached patients, 84.2% consented to take part in this study. Individuals with missing data relating to ACEs and pain assessment (*n* = 131; 4.8%) were excluded from the analyses, resulting in a final sample of *n* = 2577 patients. Of the included sample, 52.1% were female, and the mean age was 43.1 (SD: 16.2) years. The majority of patients were married or in a long-term relationship (63.6%). About half (45.5%) had received higher education or a university degree. Details are shown in Table 1.

Due to an organizational error, the ETI questionnaire was not handed out to *n* = 717 (27.8%) of these patients. Sensitivity analyses confirmed that patients who did not fill out the ETI questionnaire did not significantly differ from the remaining patients with regard to their gender (*p* = 0.96), prevalence of CP (*p* = 0.91), or depression, somatization, or anxiety scores (all *p* > 0.05). Yet, they were significantly older (42.2 vs. 45.5 years; *p* < 0.001; *d* = 0.2) and had lower MACE sum scores (0.48 vs. 0.88; *p* < 0.001; *d* = 0.3).

**Table 1:** Sociodemographic data of patients with chronic pain (i.e., symptom duration ≥ 6 months) and without chronic pain.

	CP (*n* = 813)		No CP (*n* = 1764)				
	*n*	%	*n*	%	χ^2^/t-Value	ϕ/d	*p*
Sex					2.54	0.03	0.11
Male	334	41.1%	788	44.7%			
Female	440	54.1%	903	51.2%			
Missing data	39	4.8%	73	4.1%			
Mean age (SD)	47.3	(16.3)	41.2	(15.8)			
18–30 years	151	18.6%	546	31.0%	65.94	0.17 **	<0.001
30–50 years	267	32.8%	595	33.7%			
50–70 years	271	33.3%	420	23.8%			
>70 years	67	8.2%	76	4.3%			
Missing data	57	7.0%	127	7.2%			
Relationship status					24.67	0.10 *	<0.001
Married/long-term relationship	529	65.1%	444	25.2%			
Single	154	18.9%	1111	63.0%			
Divorced	71	8.7%	97	5.5%			
Widowed	17	2.1%	16	0.9%			
Missing data	42	5.2%	96	5.4%			
Level of education					48.02	0.14 *	<0.001
School not finished	14	1.7%	29	1.6%			
Compulsory school	88	10.8%	115	6.5%			
Compulsory school and apprenticeship	325	40.0%	568	32.2%			
Higher education	202	24.8%	509	28.9%			
University degree	99	12.2%	363	20.6%			
Missing data	85	10.5%	180	10.2%			
Living situation					16.54	0.08 *	0.002
Living alone	158	19.4%	336	19.0%			
Living with partner/family	479	58.9%	989	56.1%			
Living with family of origin	38	4.7%	135	7.7%			
Living in shared apartment	41	5.0%	147	8.3%			
Missing data	97	11.9%	157	8.8%			
Living environment					1.69	0.03	0.19
Urban region	306	37.6%	710	40.2%			
Rural region	436	53.6%	900	51.0%			
Missing data	71	8.7%	154	8.7%			
Parenthood	326	40.1%	637	36.1%	6.08	0.05	0.014
Missing data	125	15.4%	239	13.5%			
Disability	83	10.2%	64	3.6%	47.25	0.14 *	<0.001
Missing data	53	6.5%	75	4.3%			

CP: chronic pain; SD: standard deviation; * small effect size, ** medium effect size, and *** large effect size.

### 3.1. Prevalence of CP and Pain Impairment

Of all included patients, 31.5% (*n* = 813) reported having CP (i.e., pain experiences of at least six months). No significant gender differences were found for the presence of CP. Patients with CP were significantly older (47.3 vs. 41.2 years; *t* = 8.7; *p* < 0.001) and reported significantly higher psychological distress (mean BSI total score: 10.8 vs. 5.6 points; *t* = 12.2; *p* < 0.001) and a higher load of trauma-related symptoms (ETI total score: 15.2 vs. 8.5 points; *t* = 9.7; *p* < 0.001).

Of all patients with CP, 15.3% (*n* = 124) did not feel impaired by their pain at all, while 29.5% (*n* = 240) reported at least mild impairment, 29.5% (*n* = 240) reported moderate impairment, and 23.7% (*n* = 193) reported severe impairment due to their pain. Pain impairment was not associated with sex (*χ*^2^ = 5.56, *p* = 0.14) or age (*r* = 0.03, *p* = 0.43). Higher pain impairment was also associated with significantly higher psychological distress (*r* = 0.28; *p* < 0.001) and more trauma-related symptoms (*r* = 0.18; *p* < 0.001).

### 3.2. Dose-Dependent Relationship of ACEs with CP

In the total sample, 64.5% (*n* = 1661) reported no ACEs at all, 29.4% (*n* = 758) reported one–three ACEs, and 6.1% (*n* = 158) reported four or more ACEs. Within the CP sample, the ACE prevalence rates were notably higher with 56.0% (*n* = 455) reporting no ACEs, 33.7% (*n* = 274) reporting one–three ACEs, and the remaining 10.3% (*n* = 84) reporting four or more ACEs. Logistic regression indicated a significant dose-dependent effect of ACEs: compared to patients with no ACEs, the odds ratios for CP increased 1.50 times (95% CI: 1.25–1.80; *p* < 0.001) for patients with one–three ACEs and 3.01 times (95% CI: 2.16–4.19; *p* < 0.001) for patients with four or more ACEs.

### 3.3. Type-Dependent Relationship of ACEs with CP

In the total sample, the most frequently reported forms of ACEs were emotional abuse (*n* = 466, 18.1%), followed by peer abuse (*n* = 356, 13.8%), physical or emotional neglect (*n* = 310, 12.0%), witnessing violence (*n* = 246, 9.5%), physical violence (*n* = 181, 7.0%), and sexual violence (*n* = 121, 4.7%). The prevalence rates were higher in the patient sample with CP, yet the relative frequency was comparable, with emotional abuse (*n* = 211, 26.0%) being the most frequent form of ACE, followed by peer abuse (*n* = 137, 16.9%), neglect (*n* = 126, 15.5%), witnessing violence (*n* = 118, 14.5%), physical violence (*n* = 92, 11.3%), and sexual violence (*n* = 60, 7.4%). Logistic regression showed that emotional abuse (OR = 1.55, 95% CI: 1.21–1.99, *p* < 0.001) and sexual abuse (OR = 1.50, 95%CI: 1.01–2.22, *p* = 0.45) were both individually associated with a 1.5-times increased likelihood of CP in adulthood, while no specific associations with CP were found for physical violence (*p* = 0.06), neglect (*p* = 0.17), witnessing violence (*p* = 0.07), or peer abuse (*p* = 0.57).

### 3.4. Timing-Dependent Relationship of ACEs with CP

Regarding the age at the time of exposure to maltreatment, we found a higher incidence of maltreatment with increasing age: 28.3% for ages 1–6 (*n* = 148), 38.4% for ages 7–12 (*n* = 201), and 39.8% for ages 13–18 (*n* = 208). About one-quarter of the patients with CP reported maltreatment during either one (26.6%) or two (25.8%) of the age periods, while almost half of the sample reported it during all three age periods (47.6%).

**Figure 1:**
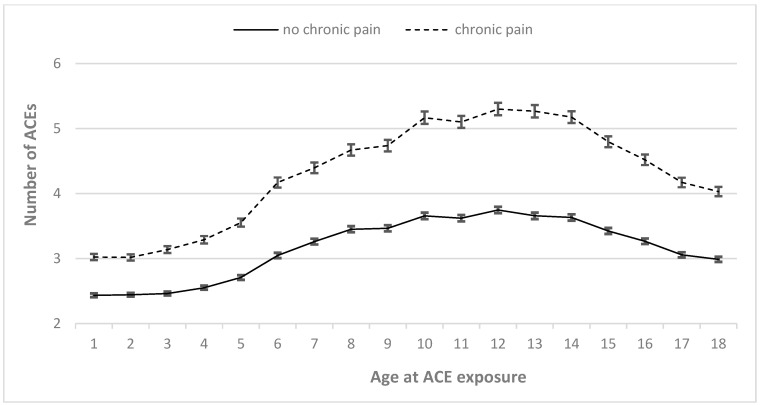
Abuse trajectories of patients with and without chronic pain: the *X*-axis represents years of age, and the *Y*-axis represents mean number and standard error of the mean (SE) of overall exposure to adverse childhood experiences (ACEs) at the corresponding year of age. Data are represented for patients with (dashed line) and without (continuous line) chronic pain (symptom duration > 6 months) separately.

The overall maltreatment trajectories showed a linear increase in childhood maltreatment with a peak reported between 10 and 14 years. There was also a significant group*age interaction effect with a small effect size (*F* = 3.31, *p* < 0.001, *η*^2^ = 0.022): patients suffering from CP reported significantly higher incidence rates of ACEs than those without CP at a similar age. As shown in Figure 1, a distinct increase in experienced abuse after the age of six years was observed in the group of CP patients.

To test for specific vulnerability periods, several predictive models were evaluated in a machine learning grid search, as briefly outlined earlier. In the first step, the age variables were provided as separate predictors (i.e., 18 variables for each year). Generally, the metric maltreatment data per year yielded superior results to the dichotomized data. The Ada Boost Classifier resulted in the best predictive model, with a weighted F1 score of 56.5%, which was found to be clearly superior to the baseline that yielded a weighted F1 score of 45.2%. The best model returned the highest values for maltreatment at six and eleven years.

**Figure 2:**
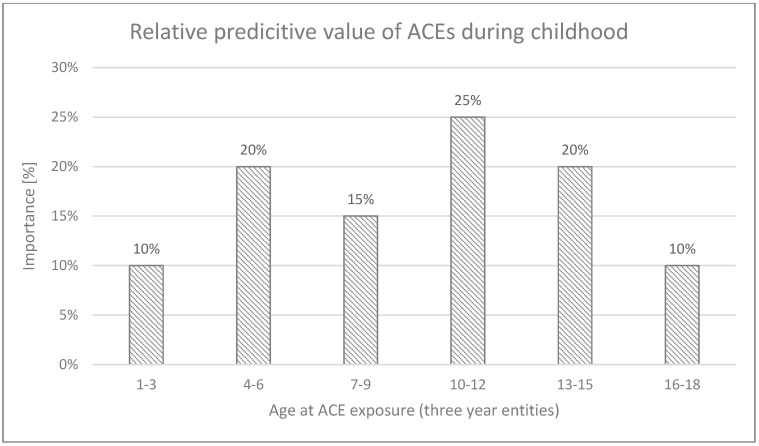
Importance of each age group in three-year entities used by the model (Ada Boost Classifier) to compute its predictions for CP. *X*-axis represent years of age clustered into three-year entities; *Y*-axis represents the relative predictive importance of the age group.

When the age groups were clustered into three-year entities, the prediction accuracy clearly improved (with a weighted F1 score of 58.6%, using the Ada Boost Classifier). Maltreatment between the ages of 10 and 12 years was identified as the strongest predictor in this model. Results are presented in Figure 2.

Clustering into two-year entities resulted in a comparable prediction rate (with a weighted F1 score of 58.5%, using the Ada Boost Classifier), while the prediction rates declined with large age clusters (weighted F1 scores: 4-year cluster: 57.0%; 5-year cluster: 56.7%; 6-year cluster: 57.3%).

## 4. Discussion

The results of our study show that CP is a highly prevalent symptom among hospital patients and that it is associated with an increased level of psychological distress. About one-third of the included general hospital patients across seven specialties reported some form of CP (i.e., experiences of pain for at least six months) and more than one-half of these patients reported moderate or severe impairment due to their experienced pain. The prevalence of CP was thus significantly higher than in the general population [1,2,3,4]. Based on the machine learning approach, maltreatment between 10 and 12 years of age was identified as the strongest predictor of CP in adulthood, thus indicating a particularly vulnerable developmental period.

ACEs were found to be prevalent significantly more often amongst patients with CP than in patients without CP, as also reported in previous studies [9,16,17]. Our data indicate a dose-dependent association between ACEs and CP, which is in line with previous pain-related research [19,20], as well as with general research on ACEs and physical health [14]. Research has shown that childhood maltreatment is associated with impaired development across multiple regulatory domains, including the nervous, endocrine, and immune systems [30,50,51,52]. While a comprehensive mechanistic account for the effect of maltreatment on pain signaling cascades and the subjective experience of chronic pain in humans is still lacking, important advances underscore the various downstream processes affected by ACEs. For instance, it has been shown in animal studies that early negative attachment experiences or early experiences of physical pain lead to the epigenetic modification of the glucocorticoid receptor gene, which influences the stress response [53] and muscular pain [54,55,56] in later life. Growing up in a chronic at-risk environment may lead to a state of heightened neurocognitive vigilance (i.e., altered threat processing, epistemic hypervigilance, and reduced support seeking), which has been argued to be a key factor for latent vulnerability to psychiatric disorders [57,58]. The biological mechanisms linking ACEs to CP involve a complex interplay of epigenetic modifications, neuroendocrine dysregulation, and structural brain alterations. Epigenetic changes, such as DNA methylation, can influence the expression of genes related to pain perception and inflammatory responses, potentially sensitizing individuals to chronic pain [59,60]. Dysregulation of the hypothalamic–pituitary–adrenal (HPA) axis, resulting in abnormal cortisol levels, further contributes to maladaptive pain processing by affecting immune function and inflammation [22]. Additionally, structural changes in brain regions responsible for pain modulation, including the prefrontal cortex, hippocampus, and amygdala, may impair endogenous pain inhibitory pathways [61,62]. The association between traumatic events and altered pain responsiveness may also largely be caused by a chronic state of hypermnesia (i.e., overactive memory) and hyperarousal. It has been demonstrated in animal models that increased levels of stress hormones (cortisol and epinephrine) can change the intracellular signaling pathway in the primary afferent nociceptive nerve fibers (nociceptors) and thus lead to enhanced and prolonged pain signals [63].

Childhood emotional abuse and sexual abuse were identified as independent ACE-specific predictors, thus indicating a potential type-specific association. Childhood emotional abuse has been associated with cortical thinning in regions relevant to self-awareness and self-evaluation, while childhood sexual abuse can lead to cortical thinning in the genital representation field of the primary somatosensory cortex [64]. Additionally, emotional abuse, witnessing violence, and sexual abuse may lead to more insecure attachment styles in adulthood [65], which are associated with increased pain sensitivity and impairment [66]. Thus, early childhood adversities may lead to increased feelings of injustice and a lack of interpersonal trust, which have been identified as risk factors for poorer physical and psychological outcomes and dysfunctional coping strategies in patients with CP [67,68]. This is especially important for patients with primarily nociceptive or neuropathic pain triggers, as it has been shown that there is a top–down effect of pain-related belief structures, such as feelings of injustice related to pain. These top–down effects may not only shape expectations about pain intensity and thus lead to more dysfunctional cognitive strategies (e.g., pain catastrophizing), as well as worse behavioral and affective responses to pain [69], but they may also lead to increased experienced pain intensity due to a negative expectation (“predictive pain”) [70].

As for the question of developmental hypersensitive periods, our data suggest a *timing-dependent relationship* of ACEs with chronic pain (i.e., at which age the abuse took place). In our sample, exposure to ACEs between 4 and 6 and 10 and 12 years was identified as the strongest predictor of CP. To the best of our knowledge, so far, no research has been published on sensitive periods of brain development in the context of CP patients. Yet, there is some preliminary evidence that the phase of early adolescence is a specifically sensitive period in brain development more generally, associated with a heightened risk for mental disorders [64]. In a comprehensive review, Teicher, Samson, Anderson and Ohashi [23] indicated an increased vulnerability for the aberrant development of the hippocampus and right amygdala during the ages of 4–6 and 10–12 years. Research has shown that pre-school years (i.e., ages 3–5) are a specifically sensitive period for hippocampal development [61], and that experiences of abuse before the age of 7 years has been found to have a disproportionate negative effect on hippocampal development and stress regulation [22,60,62]. Another study by Andersen, Tomada, Vincow, Valente, Polcari and Teicher [28] found a significantly smaller hippocampal volume in women with episodes of sexual abuse between 11 and 13 years. It has been shown that in patients with CP, a reduced hippocampal volume and other structural abnormalities are frequently discovered, which may contribute to the increased levels of anxiety and depression and deficits in learning and memory that can be observed in patients with CP [71].

Our findings highlight the significance of the early identification and treatment of affected individuals to potentially reduce the risk of CP in adulthood. Depending on co-occurring comorbidities, different evidence-based approaches—such as trauma-focused psychodynamic therapy (TF-PDT) or STAIR Narrative Therapy (SNT) [72]—are available. For adults presenting with CP and a history of trauma, integrative and multimodal therapeutic approaches are usually recommended.

While this study focuses on ACEs as risk factors for CP, it is important to acknowledge the role of protective factors that may buffer these adverse effects. Resilience, defined as the capacity to adapt positively in the face of adversity, has been shown to mitigate the impact of ACEs on various health outcomes, including CP [73]. Additionally, strong social support networks, particularly secure childhood attachments, have been associated with reduced physiological stress responses and better emotional regulation, potentially lowering the CP risk [74]. Another protective factor that has recently gained attention in psychotherapy research is the concept of mentalization—a cognitive process that enables individuals to understand and interpret internal mental states in both themselves and others. This includes the ability to reflect on one’s own thoughts, needs, emotions, wishes, and desires, as well as those of others [75,76].

The ability to mentalize—recognizing and understanding one’s own physical and mental states—may facilitate the processing of distressing experiences, enabling individuals to address emotional discomfort directly rather than relying on less effective coping mechanisms. Enhancing patients’ mentalizing capacity can thus be regarded as a critical success factor in the psychosomatic treatment of individuals with chronic physical and mental health conditions, consistent with findings from previous research [77,78,79,80], especially in patients with trauma-related symptoms [81]. It has been suggested that the experience of physical illness may be shaped by one’s level of self-awareness and insight [82].

Patient–physician interactions that emphasize mentalization may contribute to more effective pain management strategies [83]. Research has demonstrated that the quality of the patient–clinician relationship can significantly impact both treatment adherence and clinical outcomes [84,85,86]. When a physician validates a patient’s pain—acknowledging their experience and responding with empathy—it reflects the physician’s ability to mentalize the patient’s internal state. This validation signals to the patient that their experience is taken seriously and treated with respect, creating a safe environment where they can openly discuss their pain and associated challenges without fear of judgment or dismissal [87]. Such interactions foster greater trust within the therapeutic relationship. We advocate for the development of mentalization-focused guidelines and tools for healthcare providers across disciplines, as such resources could enhance the treatment of individuals with chronic pain. Further research in this area is both necessary and promising.

As to the limitations of this study, one limitation has to be seen in the retrospective collection of ACE data. A recent meta-analysis has found only limited agreement between retrospective and prospective reports of ACEs [88]. While the authors of the meta-analysis argued that retrospective assessment may be more sensitive for ACEs than prospective measures, retrospective assessments are always prone to memory bias. In general, it is assumed that retrospective self-report leads to underestimating childhood adversities; i.e., the real extent/percentage of ACEs is higher [89,90]. CP was assessed as a binary variable, with no further differentiation regarding the type or location of pain. We acknowledge that CP can stem from various etiologies and clarify that our study did not differentiate between specific diagnoses (e.g., fibromyalgia, rheumatoid arthritis). Also, no information on protective factors or resilience factors was collected. The lack of complete ETI data for the sample limits the generalizability of the regression analyses.

Data were collected from patients in the waiting areas of the participating departments, usually with other patients present. Several strategies were applied to guarantee the best possible privacy and confidentiality (see the Section 2 for details). Yet, we cannot rule out the possibility that, due to concerns about privacy, some patients either did not participate or downplayed their experiences. Finally, although we offered translated versions of the questionnaire in the languages of the major immigrant groups in Tyrol, the proportion of immigrants in our sample was small. Aside from the language barrier, sociocultural factors may also have played a role. Future studies should try to include greater cultural diversity in the research team to facilitate the better inclusion of non-native speakers. Moreover, since a cross-sectional, non-interventional study design was applied, no conclusive statements on causality can be made. One further limitation of this study is the exclusion of psychiatric inpatients. While this decision was made to mitigate selection bias and avoid overestimating the impact of ACEs on CP, it also means that our findings may not fully capture the complexities of ACE-related CP in individuals with severe mental health conditions.

Future research will have to address these limitations, mainly by employing prospective designs in order to assess causal links in the interplay between maltreatment experiences, physiological responses to heightened allostatic loads, and the subjective and biological expression of pain.

## 5. Conclusions

The results of our study showed that a considerable number of patients with CP reported ACEs. Our data indicated an association of the timing of childhood abuse with CP in adulthood. This strongly suggests that early identification and the appropriate psychosocial support for patients with ACEs is a crucial task for healthcare professionals. Fostering resilience and the treatment of psychopathology in affected patients may improve the health situation and may protect against the chronification of pain.

## Data Availability

The research data supporting this publication are stored at our institutional digital data repository for published research accessible via https://creed.lbg.ac.at/ (accessed on 24 November 2024). The data sets analyzed in this manuscript are not publicly available due to ethical and legal restrictions (data contain potentially identifying and sensitive patient information). However, pseudonymized data sets have been created for the purpose of re-use and are also accessible via creed.lbg.ac.at. Requests for access to anonymized data sets should be directed to the corresponding author.
